# Beyond spider personality: The relationships between behavioral, physiological, and environmental factors

**DOI:** 10.1002/ece3.7243

**Published:** 2021-03-05

**Authors:** Linda Hernández Duran, David Thomas Wilson, Mark Briffa, Tasmin Lee Rymer

**Affiliations:** ^1^ College of Science and Engineering James Cook University Cairns Qld Australia; ^2^ Centre for Tropical Environmental and Sustainability Sciences James Cook University Cairns Qld Australia; ^3^ Centre for Molecular Therapeutics Australian Institute for Tropical Health and Medicine James Cook University Cairns Qld Australia; ^4^ School of Biological and Marine Sciences Plymouth University Plymouth UK

**Keywords:** behavioral plasticity, behavioral type, environment, experience, ontogeny, venoms

## Abstract

Spiders are useful models for testing different hypotheses and methodologies relating to animal personality and behavioral syndromes because they show a range of behavioral types and unique physiological traits (e.g., silk and venom) that are not observed in many other animals. These characteristics allow for a unique understanding of how physiology, behavioral plasticity, and personality interact across different contexts to affect spider's individual fitness and survival. However, the relative effect of extrinsic factors on physiological traits (silk, venom, and neurohormones) that play an important role in spider survival, and which may impact personality, has received less attention. The goal of this review is to explore how the environment, experience, ontogeny, and physiology interact to affect spider personality types across different contexts. We highlight physiological traits, such as neurohormones, and unique spider biochemical weapons, namely silks and venoms, to explore how the use of these traits might, or might not, be constrained or limited by particular behavioral types. We argue that, to develop a comprehensive understanding of the flexibility and persistence of specific behavioral types in spiders, it is necessary to incorporate these underlying mechanisms into a synthesized whole, alongside other extrinsic and intrinsic factors.

## Graphical abstract

Few studies have explored the mechanisms driving the expression of personality in spiders, and what effects extrinsic and intrinsic factors (and their interactions) have on the expression of personalities. Physiological traits, particularly venom and silk, may play an important role in the expression of personalities and/or behavioral flexibility in spiders

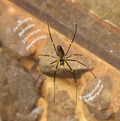

## 1 INTRODUCTION

Studies on animal personality and behavioral syndromes (also called coping styles, Table 1) have provided significant insights into how sexual selection (e.g., mate choice, sexual cannibalism; Rabaneda‐Bueno et al., 2014, and sexual conflict) and natural selection (e.g., environmental conditions, and frequency dependence; Dall et al., 2004) affect the evolution of particular behaviors (Gosling, 2001; Sih & Bell, 2008; Sih et al., 2004). The expression of repeatable behavioral traits in individuals (i.e., personality axes; Table 1), both single behaviors and suites of correlated behaviors (i.e., behavioral syndromes; Table 1), can affect the dynamics of populations and communities under different situations and contexts (Koolhaas et al., 2007; Réale et al., 2007; Wolf & Weissing, 2012). Importantly, it was often assumed that the presence of personality is indicative of a lack of behavioral plasticity (Table 1). However, it is becoming increasingly apparent that individual behavioral plasticity and/or flexibility can occur, even though behavioral consistency (i.e., personality) might constrain the limits of this plasticity (Briffa et al., 2008), and that the level of plasticity is affected by the selection pressures of particular environments, as well as how much time individuals in a population spend in particular environments (behavioral reaction norm approach; Dingemanse et al., 2010).

**TABLE 1:** Glossary of terms

Term	Definition
Aggressiveness	Degree of aggressiveness toward mates, predators, prey, and conspecifics and their response to different stimuli (i.e., aversive or novel) (Pruitt & Riechert, 2012)
Animal personality	Repeatable/consistent individual differences in behavior that are maintained over time and context (Réale et al., 2007; Sih & Bell, 2008; Sih et al., 2004). Individual style of behavioral response to stimulus or situations (i.e., the sum of all traits; MacKay & Haskell, 2015). The individual variation in behavior is related to the variation of between‐individual in the intercept of behavioral reaction norm (Dingemanse et al., 2010).
Behavioral axis	Structure for quantifying the behavioral variation within individuals or between populations in one context (i.e., each axis represents a different type of temperament or individual reaction, such as aggressive, bold, docile, active). Personality dimensions refer to populations or species (Réale et al., 2007; Mackay & Haskell, 2015)
Behavioral syndromes	Also called coping styles (see Proactive and Reactive). Correlations involving multiple behavioral and/or physiological traits shown by a set of individuals across time, contexts and situations (Sih et al., 2004; MackKay et al., 2015). For example, corticosterone concentration and boldness can be correlated within a population (Koolhaas et al., 1999)
Behavioral types	Particular combination on the behavioral axes that an individual can show and forms part of a behavioral syndrome: boldness (time to react to an aversive stimulus), aggressiveness (toward conspecifics or heterospecifics), or activity level (duration of time spent active) (Sih Bell, & Johnson, 2004; Sih et al., 2004)
Behavioral plasticity/flexibility	The ability of an animal (at the individual or population level) to change its behavior depending on prevailing environmental conditions (Briffa & Sneddon, 2016). Animals can adjust their behavior (e.g., aggressiveness) and be more plastic in different contexts and levels of behavior (Dingemanse & Wolf, 2010) Developmental plasticity is nonreversible, whereas flexibility implies reversible phenotypic change (Piersma & van Gils, 2011; Piersma & Lindström, 1997)
Boldness	Measure of the tendency of individuals to take risky behaviors (Sloan Wilson et al., 1994). Boldness is measured as the time that the individual take to react an aversive stimulus. This behavioral type is measured blowing air on the spider prosoma using a rubber‐bulb, which simulate a flying predator (Riechert & Hedrick, 1990)
Context	Domain or behavioral category where an individual performs an activity: foraging, mating, parental care, exploration of new territory, locomotion (Sih Bell, & Johnson, 2004; Sih et al., 2004)
Environment	Biotic and abiotic conditions in which different selection pressures act on an individual's phenotype and genotype (Sih Bell, & Johnson, 2004; Sih et al., 2004)
Experience	Knowledge or skills learned from previous events or situations that can be affected directly or indirectly by the environment (i.e., exposure to predators, food, soil, and space restrictions) (Johnson et al., 2015)
Ontogeny	Development of an organism over its lifetime from conception to maturation (Bosco et al., 2017)
Proactive	One type of coping style (see Behavioral syndromes) in which an animal actively responds to a stimulus (e.g., flight or fight). Often characteristic of aggressive, territorial animals (Koolhaas et al., 1999)
Reactive	One type of coping style (see Behavioral syndromes) in which an animal responds passively to a stimulus (e.g., freezing). Often characteristic of docile, nonterritorial animals (Koolhaas et al., 1999)
Situation	Condition in which a context occurs. A situation can occur at one or different points in time (e.g., breeding vs. nonbreeding season, high versus low predation risk) (Sih Bell, & Johnson, 2004; Sih et al., 2004)

While numerous studies on personality and behavioral syndromes have focused on fish (e.g., Dingemanse et al., 2010), birds (e.g., Kluen & Brommer, 2013), and mammals (e.g., Réale et al., 2009), studies on arthropods have only become prevalent within the last 20 years (Modlmeier et al., 2015; Wright et al., 2019). Particularly, spiders have become an interesting group of arthropods to study personality and behavioral syndromes (e.g., Keiser et al., 2018; Kralj‐Fišer & Schneider, 2012; Pruitt & Riechert, 2012; Sih & Bell, 2008) because they show a wide range of behavioral types (Table 1). Individuals fall along continua or axes (Table 1), such as boldness and aggressiveness (Keiser et al., 2018; Kralj‐Fišer & Schneider, 2012; Pruitt & Riechert, 2012), activity and sociability (Beleyur et al., 2015; Lubin & Bilde, 2007), which can be assessed relatively easily across different contexts and situations (Pruitt & Riechert, 2012). Ecological and behavioral hypotheses related to spider personality (Kralj‐Fišer & Schneider, 2012; Sih & Bell, 2008) can be tested across different social groups (social and solitary), clades (Mygalomorph and Araneomorph), life histories (Bonte et al., 2006), and habitats (Foelix, 2011). Spiders also show a variety of strategies for dispersal (Blandenier, 2009; Coyle, 1983), foraging (Jackson, 1992; Michálek et al., 2019) and mating (Jackson, 1992). Importantly, personality in spiders can also be studied in the context of behaviors that are common to these species, but are absent in vertebrates, such as precopulatory cannibalism (Arnqvist & Henriksson, 1997; Kralj‐Fišer et al., 2013; Rabaneda‐Bueno et al., 2008), and male emasculation during mating (Kralj‐Fišer et al., 2011). However, what makes spiders an excellent model system for testing ecological and evolutionary hypotheses related to personality is that spiders have the unique physiological traits of both venom (Cooper et al., 2015; Zobel‐Thropp et al., 2018) and silk (Blackledge et al., 2011) production, which are important drivers of behaviors and are critical for spider survival. Importantly, we are not aware of any studies that have specifically explored the relationship between both of these physiological traits and personality in spiders.

**FIGURE 1:**
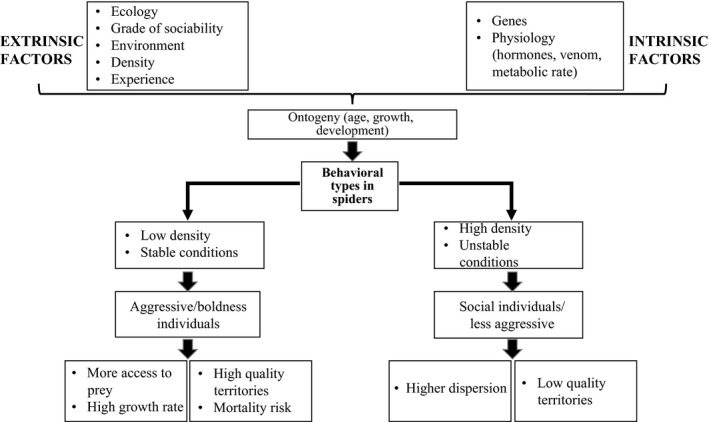
Extrinsic (e.g., environment, experience) and intrinsic (e.g., genes and physiology) factors, and their effects on ontogeny and behavioral types in spiders

To understand how and why spider personality is maintained across different contexts, we need a comprehensive understanding of what intrinsic (i.e., physiological mechanisms, such as hormones, silks and venoms; van Oers & Mueller, 2010) and extrinsic (e.g., environment) factors, and their interactions (Figure 1), directly and/or indirectly affect behaviors over the lifetime of individuals (ontogeny; Jandt et al., 2014; Kralj‐Fišer & Schuett, 2014). Although some studies investigating spider personalities have considered some underlying mechanisms independently (Bosco et al., 2017; Langenhof & Komdeur, 2018; Liedtke et al., 2015), there is still a general lack of understanding of how the various intrinsic and extrinsic factors affect the evolution of personality, as well as how they might affect behavioral plasticity (Dingemanse et al., 2010; Dingemanse & Wolf, 2010). Knowing what factors affect morphophysiological traits will provide insights into fitness and general success of spider populations (Figure 1), including how populations respond to threats, overcome challenges, and manage the costs and benefits associated with personalities (in particular, behavioral types) occurring under different conditions (Keiser et al., 2018; Kralj‐Fišer & Schuett, 2014).

Spiders use the information obtained from the environment and conspecifics over the course of their lifetime to maintain and/or modify personality (consistent individual behaviors; Fernández, 2005; Liedtke et al., 2015) and moderate the use of their biochemical weapons, namely silk and venom. Silk and venom are particularly important because they involve multiple physiological processes, regulated by numerous genes and hormones, that affect fitness (both survival and reproductive success), but also involve multiple costs (Evans et al., 2019; Nisani et al., 2007, 2012) and trade‐offs (Nisani et al., 2012; Zobel‐Thropp et al., 2018). In addition, these traits are used across different ecological contexts, such as mating, foraging, territory defense, and predation (Blackledge et al., 2011; Santana et al., 2017; Schendel et al., 2019). It has been suggested that personality (Table 1) could reflect animal life‐history trade‐offs (Wolf et al., 2007). Given that venom and silk production are both costly and directly related to prey capture (and thus the rate of food intake), it seems likely that variation between individuals in these specific physiological traits could covary with behavioral differences (Michálek, Řezá, Líznarová, Symondson, & Pekár, 2019). Thus, investigating links between venom and silk production and behavior seems like an obvious approach toward increasing our understanding of personality in spiders. Moreover, understanding these potential links in spiders could provide general insights into how life‐history trade‐offs could underpin animal personality.

Therefore, in this review, we first offer some examples of how environmental conditions, previous experience, and ontogeny induce changes in behavioral types in spiders. Then, we discuss some of the physiological traits and mechanisms, specifically hormones/neurotransmitters, and silk and venom production in relation to personality, areas that are understudied, but which we posit offer fruitful ideas for future study. Connecting these extrinsic and intrinsic factors that may drive the presence or absence of personalities in spiders will contribute to a greater understanding of the evolution and maintenance of behavioral types and behavioral syndromes in spiders, and more broadly.

## 2 THE ROLE OF ENVIRONMENT, EXPERIENCE, AND ONTOGENY IN SHAPING SPIDER PERSONALITIES

The majority of work conducted on personality in spiders has focused on boldness and aggressiveness (e.g., Keiser et al., 2018; Kralj‐Fišer & Schneider, 2012; Pruitt & Riechert, 2012), and activity and sociability (e.g., Beleyur et al., 2015; Lubin & Bilde, 2007). Importantly, behavioral types and personalities can be influenced both directly and indirectly by a wide range of extrinsic factors and proximate mechanisms (Table 2).

**TABLE 2:** Factors inducing changes in behavioral types in spiders, including the physiological traits, body state variables, and behaviors that were tested in various studies

Species	Behavior/s	Factors				Reference
		Environmental conditions	Experience	Ontogeny	Physiological and body state variables	
Agelenidae						
*Agelenopsis aperta*	Antipredator behavior; Agonistic behavior	X	–	–	–	Riechert and Hedrick (1993)
*Agelenopsis lisa*	Aggressiveness; Foraging; Exploration	–	–	X	Life‐history stage; Sex	Bosco et al. (2017)
Araneidae						
*Larinioides sclopetarius*	Aggressiveness; Boldness (plasticity vs. personality)	X	–	X	Body condition; Sex	Kralj‐Fišer and Schneider (2012)
*Parawixia bistriata*	Foraging	X	–	–	Fecundity	Fernández (2005)
Eresidae						
*Stegodyphus sarasinorum*	Sociability (personality task differentiation)	–	–	X	Body condition; Nutritional state	Parthasarathy et al. (2019)
Lycosidae						
*Lycosa hispanica*	Aggressiveness; Voracity	–	–	X	Body mass; Sex	Rabaneda‐Bueno et al. (2014)
*Pardosa agrestis*	Activity; Voracity	–	–	X	Life‐history stage	Rádai et al. (2017)
Philodromidae						
*Philodromus albidus*	Aggressiveness; Boldness	–	–	–	Possibly state dependent?	Michalko et al. (2017)
Pisauridae						
*Dolomedes triton*	Foraging; Antipredator; Voracity	–	–	X	Body condition; Fecundity	Johnson and Sih (2005, 2007)
Salticidae						
*Cosmophasis umbratica*	Foraging	–	X	–	–	Chang, Teo, et al. (2017)
*Eris militaris*	Activity; Aggression; Boldness; Voracity	X	**–**	**–**	Body condition; Body size; Sex	Royauté et al. (2014)
*Marpissa muscosa*	Exploration; Social; Learning	X	X	–	Life‐history stage; Sex	Liedtke and Schneider (2017); Liedtke et al. (2015)
*Portia labiata*	Foraging; Aggressiveness	–	X	–	–	Chang, Ng, et al. (2017); Chang, Teo, et al. (2017)
Theridae						
*Anelosimus studiosus*	Boldness (flexibility and individual consistency); Aggressiveness	X	–	X	Nutritional state; Reproductive state; Hormones	Watts et al. (2015); Price (2016)
*Latrodectus hesperus*	Dispersal; Aggressiveness	X	–	X	Nutritional state	Halpin and Johnson (2014); Johnson et al. (2015); DiRienzo and Montiglio (2016)

### 2.1 Environment and experience

Environmental and ecological factors (Figure 1), such as social context (Webster & Ward, 2011), abiotic conditions (Liedtke et al., 2015; Watts et al., 2015) and food availability (Riechert & Hedrick, 1993), are known to affect the expression of behavioral types across a variety of vertebrate and invertebrate taxa. These environmental and ecological factors have important consequences on individual life‐history traits including fertility, fecundity (Réale et al., 2007), metabolic rate (Réale et al., 2010), and body size (Johnson & Sih, 2005; Settepani et al., 2013; Vollrath & Rohde‐Arndt, 1983). Some of these factors have also been shown to affect the expression of behavioral types in spiders. For example, solitary jumping spiders, *Marpissa muscosa*, reared in poor conditions are more reactive to threat stimuli, are less willing to attack prey, and explore new environments, compared to spiders reared under semi‐natural conditions. Possibly this occurs because of an absence of natural selection pressures (e.g., predation, conspecific interactions, complex environments) that allow individuals to maintain a behavioral type, or interactions with conspecifics (i.e., family effects) that allow individuals to develop a particular behavioral type, which influences developmental plasticity (Liedtke et al., 2015). Similarly, environmental insecticide treatment leads to a break down in behavioral syndromes (consisting of activity, aggression, boldness, and voracity) of the jumping spider *Eris militaris* via disruptions specific to the activity of the spiders *(*Royauté et al., 2014). Finally, nonbrooding female *Anelosimus studiosus* were found to shift from shy to bold at night, whereas brooding females remained bold regardless of time of day, possibly because brooding females are preparing to increase foraging behavior, avoid predators, and protect spiderlings after birth (Watts et al., 2015).

Broadly, it has been suggested that specific environmental conditions can promote the evolution of intraspecific variation in behavioral types because these local conditions expose individuals within a population to selection pressures that differ to other populations (Sih & Bell, 2008). As a result, the composition of the group in relation to a behavioral type such as aggressiveness can affect the survival rate, as is seen in *Zygiella x‐notata* in urban environments (Kralj‐Fišer et al., 2017). This is because intraspecific variation in behavioral types provides the “raw material” on which natural selection can act and equates to the presence of a diversity of behavioral strategies that can be used to exploit new environments in different ways (Kralj‐Fišer & Schneider, 2012; Sih & Bell, 2008; Sih et al., 2004). Populations of individuals that show differences in behavioral types also have a better chance of coping with environments that have experienced rapid transformations, such as when a habitat changes rapidly because of anthropogenic activities (Sih, 2011).

In general, the behavioral types expressed under specific environmental conditions can also lead to changes in distribution (Sih et al., 2012), dispersion (Cote et al., 2010) and the ability to colonize new habitats (Duckworth & Badyaev, 2007; Hudina et al., 2014; Rehage et al., 2016). Particular life‐history traits, such as fast growth and short reproduction, as well as personality traits, such as aggressiveness or boldness, could explain high rates of colonization of new environments (Fogarty et al., 2011; Kralj‐Fišer & Schneider, 2012), and displacement of native species (Fogarty et al., 2011; Wolf & Weissing, 2012). Spiders are well known for these dispersal capabilities (Parthasarathy & Somanathan, 2020) and the ability to colonize new environments through the expression of different behavioral types. For example, a mix of bold and aggressive individuals in a population of *Larinioides sclopetarius* promotes the spread of the population in urban environments (Kralj‐Fišer & Schneider, 2012).

Ecotypic variation in individual behavior can also emerge in response to environmental adaptation (Riechert et al., 2001). For example, some behavioral types in *L. sclopetarius* that are expressed in a particular habitat can be inherited (Kralj‐Fišer & Schneider, 2012). The expectation would be that offspring from populations that have experienced different selection pressures would exhibit differences in prey capture, territory defense, and antipredator responses. This has been demonstrated in whip spiders *Phrynus longipes* (an arachnid related to spiders), where individuals from cave environments were more vigilant, less active, and less likely to escalate to aggression than individuals from environments on the surface, most likely because of variation in predation pressure, which drives selection for different behavioral types in different environments (Chapin, 2015). A similar pattern has been observed in the colonial spider *Parawixia bistriata*, where spiders from low resource environments show higher levels of group foraging and feeding, and greater plasticity in behavior than individuals from high resource environments (Fernández, 2005), suggesting prey availability is exerting strong selection pressure on this species' behavior.

Personalities and behavioral syndromes may manifest under particular environmental conditions (Pinter‐Wollman et al., 2012) because conditions experienced by individuals during their ontogeny likely trigger specific physiological cascades (e.g., differential hormone expression; epigenetic regulation) that regulate the expression of particular behaviors that aid survival under those conditions (Sih, 2011; Stamps & Groothuis, 2010). We might expect behavioral syndromes to emerge under stable and predictable environmental conditions because selection pressures acting on individuals from these populations lead to local adaptation, which will persist over generations (Rymer et al., 2013). Particular personalities and/or behavioral syndromes, such as boldness‐aggressiveness, emerge under particular social conditions. For example, in black widow spiders *Latrodectus hesperus*, a higher number of social interactions during early life were associated with a fast dispersal style, mostly likely because social interactions indicate potential future competition, cannibalism or inbreeding, necessitating a greater need to disperse (Johnson et al., 2015).

In contrast, behavioral flexibility might be more advantageous to allow organisms to respond to rapidly changing environmental conditions. In *L. sclopetarius*, behaviors related to foraging and aggression in novel environments could have lower heritability, at least in the first generation, whereas intrasexual bold‐aggressive behaviors have higher heritability, suggesting that plasticity could play a role in the success of these species in urban environments through negative frequency‐dependent selection, which acts to generate genetic polymorphisms for aggressiveness and boldness at the population level (Kralj‐Fišer & Schneider, 2012). Both behavioral plasticity and consistency facilitate the colonization of new environments; some evidence for this is seen in orb‐weaving spiders, where behavioral flexibility and behavioral consistency (i.e., aggressiveness) of spiders in urban environments increase the survival in high density conditions (Kralj‐Fišer et al., 2017).

Previous experience in a specific environment has also been broadly suggested to promote changes in individual behaviors to be flexible and adaptive (Figure 1), and depending on the circumstances, these changes in individual variation in behavior, potentially mediated by learning, can either persist over time or shift dynamically with environmental conditions (Dingemanse & Dochtermann, 2013; Sih, 2011). Specifically, for the social spiders *Stegodyphus dumicola* and *A. studiosus*, several reasons have been suggested for an individual's previous experience to affect the plasticity of colony behaviors. Firstly, individuals differ in their ability to respond to new conditions, stimuli or threats, which in turn influences how they respond to these stimuli (Wright et al., 2016). These consistent individual‐level responses then influence group‐level behavioral responses (Wright et al., 2016). Secondly, individuals may perform specific tasks, and continual experience with the task influences the tasks performed by others. Thirdly, individuals differ in their behavioral types in the colony; these individual differences maintain the behavioral stability of the entire colony (Jeanson & Weidenmüller, 2014; Parthasarathy et al., 2019). However, depending on the behaviors (e.g., collective foraging behavior in *S. dumicola*), changes in local conditions and density, colonies in different populations need time to adapt to other changes and to adjust their behaviors to new conditions (Keiser et al., 2014).

### 2.2 Ontogeny

Throughout their lifetime, animals undergo a sequence of physiological changes in response to environmental changes and experience, which affect the development and expression of behavioral and morphological traits (Bosco et al., 2017). Developmental changes in behavior and physiology that spiders experience from juvenile stages to adulthood can provide us with a better understanding of sexual selection, sexual dimorphism, and sexual conflict, and how apparently nonadaptive behaviors can be maintained in spider populations (Elgar & Schneider, 2004; Johnson & Sih, 2005; Santana et al., 2017). The variation in behavioral types observed over an individual's ontogeny, and the reasons why these may only be present at particular points in time, may allow us to determine what conditions (internal, external and experience) promote and maintain personalities and behavioral syndromes in spider populations. However, adjustments in behavior come with associated costs. For example, while aggressive spiders under low population density tend to have higher quality territories, they also suffer higher mortality (Fogarty et al., 2011; Keiser et al., 2018; Kralj‐Fišer et al., 2017; Réale et al., 2007; Riechert & Hedrick, 1993; Sih et al., 2015). As a consequence of how ontogenetic effects impact the expression of particular behaviors (Langenhof & Komdeur, 2018), it is also necessary to understand how ontogenetic factors affect individual personalities and, ultimately, population‐level behavioral syndromes (Bosco et al., 2017; Branch et al., 2015; Sih & Bell, 2008). Furthermore, it is important to consider how different behavioral types, exhibited over the course of an individual's development, are affected in response to environmental changes (Langenhof & Komdeur, 2018).

Consistent individual variation in behavior between adults (personality) and its correlation across different contexts (behavioral syndrome) is not necessarily present during all life stages in some animals (e.g., zebra finches, *Taeniopygia guttata*, Wuerz & Krüger, 2015), including spiders (Bosco et al., 2017; Parthasarathy et al., 2019). Early life stages are often more sensitive to environmental conditions, such as temperature, population density and food availability, which can affect the presence or absence of behavioral syndromes. For example, boldness in the desert funnel‐web spider, *Agelenopsis lisa*, tested across different contexts (foraging, placement in a new environment and response to predation) was not consistent across different ontogenetic stages, apart from the penultimate stage (Bosco et al., 2017). The aggressiveness‐boldness syndrome observed in these spiders during the penultimate stage is commonly seen in males close to maturity because they need to increase their mass, which is associated with increased mating success as adults (Bosco et al., 2017). Similarly, the repeatability of boldness and aggressiveness declines over time in subadult *S. sarasinorum*, but this is not related to body condition or nutritional state, suggesting underlying ontogenetic effect(s) on the development of personality (Parthasarathy et al., 2019).

Changes in behavior require time and energy at both neurological and physiological levels (e.g., rewiring neural paths or changing metabolism), so individuals should maintain an intermediate strategy to balance energy and time costs (Bell, 2007a). Consequently, individuals may not be able to exhibit optimal behaviors in every context, which could lead to suboptimal behaviors in different environments, leading to the establishment of conflicts and trade‐offs (Bell, 2007b; Sih Bell, & Johnson, 2004; Sih et al., 2004). However, nonadaptive behaviors, such as sexual cannibalism (e.g., garden spider *Araneus diadematus*, Elgar & Nash, 1988; orb‐weaver spider *Argiope aemula*, Sasaki & Iwahashi, 1995), and their incorporation into behavioral syndromes (e.g., voracity and conspecific aggressiveness in foraging and mating) can be explained when ontogeny is taken into consideration (i.e., the aggressive spillover hypothesis; Arnqvist & Henriksson, 1997). Precopulatory sexual cannibalism in spiders occurs when adult females cannibalize males before mating. However, this behavior is correlated with aggression based on the general feeding voracity developed by juveniles (Elgar & Schneider, 2004; Johnson & Sih, 2005). Aggression toward conspecifics is present over all spider developmental stages and is positively correlated with precopulatory sexual cannibalism in adults. Johnson and Sih (2005) also found that foraging voracity is positively correlated with boldness toward predators in fishing spiders, *Dolomedes triton*, with individuals emerging from water faster when they experience an aversive stimulus. In addition, although precopulatory sexual cannibalism in *D. fimbriatus* can lower reproductive success, females have a competitive advantage by increasing their growth rate and fecundity (Arnqvist & Henriksson, 1997), showing how nonadaptive behaviors in one context may persist over time (Riechert & Hedrick, 1993).

## 3 PROXIMATE MECHANISMS

Currently, most studies in spiders have focused on determining what extrinsic factors (e.g., environmental conditions) affect behavioral types and personalities throughout the life history of different species (Langenhof & Komdeur, 2018; Liedtke et al., 2015; Parthasarathy et al., 2019). These studies have not included proximate mechanisms (i.e., physiological traits, such as hormone concentrations, silk production, venom composition, metabolic rates, energy reserves, and immune responses) that can affect personality (Sih Bell, & Johnson, 2004; Sih et al., 2004) and play a key role in a spider's development and survival. For example, in some species of myrmecophagous spiders, use of silk and venom as hunting strategies will depend on prey specialization and adaptation to exploiting alternative prey (Michálek, Řezá, et al., 2019). The role that these proximate mechanisms play in the maintenance of individual behavioral differences is important because these mechanisms can induce a myriad of changes during different ontogenetic stages, and over different contexts and situations (Briffa & Sneddon, 2016; Sih et al., 2014). These physiological variables comprise both morphological and physiological traits and can affect interspecific and ecological relationships (e.g., sex ratio, density of individuals, predators, competitors, and parasites) that maintain behavioral differences between individuals (Sih et al., 2015).

### 3.1 Hormones and personality in spiders

The endocrine system, and its effects on individual differences in behavior, has been poorly studied in spiders. However, neuroendocrine traits allow us to understand how an animal behaves in a specific situation or in response to a threat, and how physiological and behavioral traits might be correlated (coping styles; Briffa & Sneddon, 2016). Biogenic amines, namely neurotransmitters, hormones, and neuromodulators, act on the central and peripheral nervous systems, allowing arthropods to respond to different stimuli (Roeder, 2005). For example, when an individual is exposed to a threat stimulus, this triggers a response that regulates the release of these biogenic amines that then increase the individual's aggressive or defensive behaviors (Bengston & Jandt, 2014; Jeanson & Weidenmüller, 2014; Roeder, 2005). Additionally, these biogenic amines can be affected by genetic and environmental conditions, mediating changes in different behaviors and personality (Edenbrow & Croft, 2013).

In arthropods in general, the concentration of biogenic amines, such as octopamine and serotonin (Roeder, 2005; Roeder et al., 2003), can influence a wide range of behaviors, including aggression, territory defense and escape behaviors (Jones et al., 2011). For example, in orb‐web spiders, *L. cornutus*, increased octopamine concentrations reduce the time to respond when a spider is exposed to a predator or other aversive stimulus (Jones et al., 2011). Although hormones may act differently between species, they can also mediate differences in behavioral tendencies, like aggressiveness, by inducing changes in activity level, or by either reducing or increasing aggression (e.g., *A. studiosus, L. cornutus*, Jones et al., 2011; Price, 2016).

Hormones also have direct and indirect effects on the immune system. For example, in cellar spiders, *Physocyclus dugesi*, juvenile hormone (acyclic sesquiterpenoids) down‐regulates the immune response during mating (Calbacho‐Rosa et al., 2012). Lower concentrations of juvenile hormone are associated with lower aggression in honey bees, *Apis mellifera* (Pearce et al., 2001), and aggressive behavior is also associated with down‐regulation of the immune response, potentially mediated via juvenile hormone, in other arthropods (e.g., beautiful demoiselle, *Calopteryx virgo*, rubyspot damselfly *Hetaerina americana*, Contreras‐Garduño et al., 2006; Contreras‐Garduño et al., 2009). Neurohormones also regulate different processes (e.g., ontogenesis, sexual maturation, and ecdysis) that may impact the expression of behavior in spiders in general (Sawadro et al., 2017), and these hormones may also impact other physiological properties, such as venoms and silks, which then further impact behavior.

### 3.2 Venom properties and personality in spiders

In venomous animals, venom production involves high metabolic costs (Evans et al., 2019; Nisani et al., 2007, 2012), but it also plays an important role in survival (Cooper et al., 2015). Venom production and composition, and their associated costs, are known to be affected by different extrinsic factors, including diet, habitat, climate (Boevé et al., 1995; Cooper et al., 2015), season (Atkinson & Walker, 1985), niche specialization (Bergmüller & Tab orsky, 2010; Michalko et al., 2017; Michalko & Pekar, 2014), and predation risk (Gangur et al., 2017). For example, in the funnel‐web spider, *Atrax sutherlandi*, the venom yield from spiders collected in winter, is higher than that collected in autumn, suggesting temporal variation in venom production within the species (Wong et al., 2016).

In addition, differences in the quantity and quality of venom are affected by different intrinsic factors (e.g., hunger level, Hayes, 1993; life‐history stage; Herzig, 2010), metabolic rate (Kowalski & Rychlik, 2018), hormone concentration (Gomes & Palma, 2016; Lira et al., 2017; Zhang et al., 2017), body size (Fox, 2018; Rocha‐e‐Silva et al., 2009), genes (Case well et al., 2013; Hargreaves et al., 2014), sex (Zobel‐Thropp et al., 2018), and/or ontogeny (Boevé et al., 1995). For example, sexual dimorphism in venom profiles is seen in the orb‐weaver, *Tetragnatha versicolor*, which is thought to play a role in sexual communication (Zobel‐Thropp et al., 2018), while venom composition in the tarantula *Phlogius crassipes* changes during development from the juvenile stage through to adulthood and continues to change throughout adulthood (Santana et al., 2017).

However, how these extrinsic and intrinsic factors interact to affect morphology, physiology, and behavior are complex, and potentially species‐specific. For example, sex and development affect venom yield in the rainforest tarantula, *Coremiocnemis tropix*, whereas not the availability of food (Herzig, 2010). Orb‐web spiders, *Tetragnatha versicolor,* show sex differences in venom properties, ecological functions, and behaviors when they are threatened (Zobel‐Thropp et al., 2018). In the funnel‐web spider, *A. robustus*, the venom in males has higher mammalian neurotoxin activity than in females, but the toxins that cause the envenomation syndrome are only present during the male adult stage (Gray & Sutherland, 1978; Herzig et al., 2020; Wilson, 2016). While ecological and biological factors can influence these developmental changes in species in general (Sih et al., 2015), we do not yet know what specific intrinsic states trigger these changes in *A. robustus* males.

Venom is used across multiple ecological, contexts including mating, territory defense, feeding/foraging, and predator deterrence (Cooper et al., 2015; Schendel et al., 2019). The costs associated with the production and use of venom should be compensated by using the venom in an optimal way through modulation of the quantity, and/or potentially the composition, of venom toxins (venom optimization hypothesis or venom metering, Boevé et al., 1995; Cooper et al., 2015; Morgenstern & King, 2013; Nelsen et al., 2014; Schendel et al., 2019; Wigger et al., 2002). The costs associated with venom use can be direct, such as energy used in the production and storage of toxins, and/or indirect, such as in a reduced capacity to capture prey or deter predators (Evans et al., 2019). For example, in *Cupiennius salei*, the volume and toxicity of venom is not regenerated at equal rates; 50% of the volume of venom can be regenerated in one day, but the toxicity of the venom can take days or weeks to completely regenerate (Boevé et al., 1995). The spiders can compensate for some of these costs by optimizing the use of venom in relation to the amount of venom available in their glands (Wullschleger & Nentwig, 2002). In addition, they show differences in prey capture behavior, using multiple strategies when different prey are encountered. *C. salei* use only a small amount of venom on small prey, such as crickets, but expend more venom on larger prey (Boevé et al., 1995; Wigger et al., 2002). Similarly, the wandering spider, *Phoneutria nigriventer,* uses its chelicerae to cause mechanical damage to small prey and only use venom when the prey is large (Schenberg & Pereira Lima, 1978). Finally, the orb‐weaver, *Argiope argentata,* uses short bites when prey is small and long bites when prey is larger (Robinson, 1969).

Unfortunately, the quantity and composition of venom, as well as the physiological costs and the time taken to regenerate the venom (recovery period), have not been studied in relation to personality and behavioral syndromes in spiders. In social spiders, aggressive individuals interact more intensely with both predators and prey than do docile individuals (Riechert, 1993), but we do not know what and/or how other traits might change during these interactions. We speculate that aggressive spiders would experience a higher metabolic cost, as occurs in scorpions *Parabuthus transvaalicus* (Nisani et al., 2012), because this is associated with a higher concentration and quantity of venom required when subduing prey. It would be interesting, to test if aggressive spiders use different toxins during intraspecific competition, as occurs in the polyps of the aggregating sea anemone *Anthopleura elegantissima* (Macrander et al., 2015) and ants of the genus *Monomorium* (Westermann et al., 2015). Aggressive anemone polyps show a higher quantity of a particular type of gated potassium ion channel (toxins/Kunitz‐type protease inhibitor and type II acrorhagins; Macrander et al., 2015), whereas ants using venom to withstand attack from the invasive Argentine ant *Linepithema humile* show higher concentrations of toxins compared to populations of ants that do not live in close proximity to these invasive ants (Westermann et al., 2015). In the funnel‐web spider *A. robustus*, males are more aggressive and more prone to attack when they are provoked than females, which could be correlated with higher venom toxicity (Mullen & Vetter, 2019). This behavioral type and venom toxicity likely provide a survival advantage for males, as males are more exploratory because they have to search for sedentary females, deter predators, subdue prey and reduce conspecific competition (Stoehr & Kokko, 2006). However, the associated trade‐offs of higher aggression and venom toxicity might also include greater exposure to predators, higher metabolic costs, and lower immune efficiency (Nisani et al., 2012; Zobel‐Thropp et al., 2018). Thus, it is necessary to study the different functions and properties of venom between males and females, and their link with intrinsic and extrinsic factors that shape behavioral types.

Multiple questions can be asked about particular patterns of relationships between behavior, venoms, and their ecological functions. Intraspecific variation in venom composition and regeneration has been reported in funnel‐web spiders, *Hadrochyne infensa*, from Toowoomba and Fraser Island in Australia (Palagi et al., 2013). However, we should consider if the regeneration of venom is faster in aggressive individuals. That is, does venom volume and/or composition differ consistently across individuals? Similarly, are the metabolic costs higher for one particular behavioral type, or do different behavioral types adjust their behaviors to compensate for a reduction in venom volume, and is this compensated for in some way? For example, aggressive individuals could waste resources, expelling more venom when a predator is present, but these individuals might have a better ability to colonize new environments (Johnson et al., 2015; Kralj‐Fišer & Schneider, 2012). Similarly, spiders might balance the costs of performing a particular behavior across different contexts, such as mating and foraging. In hairy desert scorpions, *Hadrurus arizonensis*, males use a soft movement of the telson to sting females during courtship, and this movement is also used when scorpions immobilize their prey (Coelho et al., 2017; Tallarovic et al., 2000). Understanding individual variation in venom composition and the costs associated with its use could explain how some personalities are maintained and evolve in different spider species. Likewise, linking behaviors and physiological traits will allow us to explore the ability of individuals to be flexible in response to changing environmental conditions.

### 3.3 Silk properties and spider personality

Silk is a key feature of a spider's biology. The evolution of silk properties and its uses in spiders is related to selective pressures that affect spinning behaviors, ecology, and the physiological production of silk (Vollrath, 1999). Different taxonomic groups of spiders have shown modifications in the use of silk, and the variation is linked to the type of habitat, prey capture strategy, predator deterrence, and mating (Blackledge et al., 2011; Garb, 2013; Starrett et al., 2012). Arguably, the most important factor affecting web properties and architecture is the type of prey that the spider catches, which can change as the spider ages (Sensenig et al., 2011). Mygalomorphs and Mesothele have a morphologically simple and uniform set of silk glands that are related to a sit‐and‐wait strategy for subduing prey (Sanggaard et al., 2014; Starrett et al., 2012). On the other hand, Araneomorph orb‐weavers represent the widest diversity of silk types that are functionally distinct (Garb, 2013). Seven to eight glands (Blackledge & Hayashi, 2006; Garb, 2013) produce functionally different types of silks that are used to (a) build the frame, radii and draglines of the web (major ampullate), (b) construct the temporary capture spiral of the web (minor ampulla), (c) make the core fiber of the capture spiral (flagelliform), (d) produce sticky droplets that coat the capture spiral (aggregate glands), (e) make the outer egg case (tuniliform; specific to females), (f) wrap prey and produce the soft inner egg case (actiniform), and (g) secure fibers to substrates (pyriforms). These glands vary in morphology and number between Araneomorph species, which is consistent with the evolution of function, material, mechanical properties of the silk, and the wide diversity of habitats and behaviors that Araneomorphs exhibit (Blackledge et al., 2011; Garb, 2013; Vollrath, 1999).

Spiders use silk during different phases of their life cycle, across a variety of ecological contexts, and for multiple functions (Garb, 2013). Variation in silk production can also differ during development (Garb, 2013; Moon & Kim, 2005). For example, in male orb‐weaving spiders, their flagelliform and aggregate glands are lost when they molt to adults (Garb, 2013; Moon & Kim, 2005). Similarly, in the orb‐weaver spider, *Neoscona arabesca*, silk properties in webs change with development, where the strength, toughness and web performance change as the spider grows (Sensenig et al., 2011). Silk production and use also varies within species (Sensenig et al., 2011) and between the sexes. In Araneomorph spiders, males produce fewer types of silk than females (Garb, 2013) due to the loss of silk glands (Moon & Kim, 2005). Males also have epiandrous glands that, along with actiniform glands, are used to build the sperm web where sperm is deposited prior to being transferred to the pedipalps for mating (Moon & Kim, 2005).

However, there are some constraints and costs associated with silk production and use (Blackledge et al., 2011; Craig et al., 1999). Synthesizing amino acids is one constraint for silk production, and the amount of energy spent in this process will depend on the metabolic pathway that the spider uses (Blackledge et al., 2011). Additionally, behavioral costs of spinning involve energy consumption, but this differs between orb‐weavers, which use viscid glue in their silk to capture prey; in contrast, cribellate spiders produce silk fibers without viscid glue (Blackledge et al., 2011). Cribellate silk is more expensive to produce and demands more time in construction in contrast to webs built by orb‐weavers (Blackledge et al., 2011; Craig, 2003). Orb‐weaver spiders recycle amino acids from old webs and use them to generate new silk. Recycling silk reduces the costs of spinning by 32% (Craig, 2003). Similarly, the costs of web relocation include exposure of spiders to increased predation risk (Nakata & Ushimaru, 2013), and relocation is time‐consuming because it requires that the spider samples prey in a different location for many days until it finds a good location to build the web (Blackledge et al., 2011).

Spiders can adjust their spinning behavior, biochemical composition, and web architecture depending on different factors, such as prey abundance, predation risk, and environmental conditions (Blackledge et al., 2011; Craig et al., 2000; Vollrath, 1999; Vollrath & Selden, 2007). For example, black widow spiders spin two different types of webs depending on prey abundance: starved spiders produce a classic cob‐web, while satiated spiders change their behavior to produce an elaborate network made of supporting threads (Blackledge & Zevenbergen, 2007). Silk also protects spiders against predators. For example, the orb‐weaver, *N. arabesca*, adds items such as leaves, silk stabilmenta (web decorations), or barriers to reduce predation risk (Sensenig et al., 2011). However, the behaviors performed to avoid predation can have long lasting impacts on the fitness of both an individual and a population. For example, the social spider, *S. dumicola*, produces special cribellate silk to make a tangled silk barrier during attacks from ants (Henschel, 1998). However, this type of silk is commonly used to repair and construct the web for prey capture and is costly to produce over extended periods of time and can contribute to the spread of a fungal disease (Henschel, 1998).

Web‐hunting strategies play a role in the type of silk used, as well as the silk's properties (Sensenig et al., 2011). However, other groups of spiders use different strategies for hunting. Instead of investing energy building a capture web, pirate spiders (Araneae, Mimetidae), jumping spiders (*Portia* spp.), and *Poecilochroa senilis* (Araneae, Gnaphosidae) invade the webs of other spiders, using aggressive mimicry or stealthy approach to capture the resident spider (Jackson & Whitehouse, 1986; Li & Jackson, 1996; Michálek et al., 2019). Other spiders, including mysmenids and theridiids (e.g., *Argyrodes*), steal the silk (kleptoparasitism) and prey from other resident spiders without being detected (Tso & Severinghaus, 1998). These araneophagus spiders engage in risky behaviors to use another spider's web and capture the host, which comes with a high cost of being predated. These spiders can assess how dangerous the targeted spider prey is and make decisions on whether to attack the host spider depending on its size (Chang et al., 2017).

There has been some consideration of the relationship between personality and silk production in spiders. How species might choose prey, or which type of prey to target, could be associated with specific behavioral types as well as cognitive types/styles (behavioral types related to decision making). It has been proposed for animals in general that bold, aggressive, exploratory, and active individuals tend to be faster making decisions related to hunting (Sih & Del Giudice, 2012). This is supported by studies of jumping spiders, *Portia labiata*, where individuals show differences in aggressiveness and speed of prey‐choice decision, with aggressive individuals making decisions faster than docile ones (Chang et al., 2017). Furthermore, the use of silk depends on personality composition, social organization and collective behavior in colonies of the social spider *S. dumicola*. When attacked by ants, bolder spiders participate in cribellate silk making, while shy individuals carry out body attack and leg immobilization (Wright et al., 2016). Consequently, colonies with a mix of bold‐shy personalities show better defense of their webs than monotypic colonies (Wright et al., 2016).

Vollrath and Selden (2007) made the broad observation that individual spiders vary not only in specific morphological and anatomical traits, but also in the way they use different silks (i.e., inter‐individual variation in behavior). Given that spinning behavior changes according to the conditions in which spiders are exposed (Vollrath & Selden, 2007), we suggest that changes in spinning behavior could also be interlinked with behavioral repeatability or consistency (i.e., personality), which likely impacts spider survival. This is supported by findings in jumping spider, *M. muscosa*, reared under poor conditions and changed prey availability, where individual variation in behavior is not consistent (Liedtke et al., 2015). Consistency and/or behavioral plasticity likely differs between species. For example, compared to *M. muscosa*, *S. sarasinorum* individuals show behavioral consistency and plasticity in prey capture when availability of prey is low (Beleyur et al., 2015). If individual variation in behavior is adaptive, and there is predictability in environmental conditions, then there is likely a balance between consistency and flexibility, allowing individuals to change the use of resources, such as silk, to respond to changes in conditions (Watts et al., 2015).

### 3.4 How are hormones, venoms, and silks related?

Establishing a connection between hormones (e.g., octopamine, serotonin and juvenile hormone) that affect the expression of behavioral types (e.g., aggressiveness), and the use and modulation of venom in a particular context, would aid our understanding of the evolution of personalities in spiders. Recently, Undheim et al., (2015) showed that the evolution of one class of venom peptides in Araneomorph spiders and centipedes was derived from an ancient family of neuropeptide hormones that subsequently became a toxin through structural adaptation. This suggests that hormones and venoms may be closely interlinked and, perhaps, work synergistically in affecting behavioral expression. However, this would require considerable testing to elucidate if these relationships do occur.

Venom and silk are used to capture prey and deter predators (Sensenig et al., 2011). Often these biomolecules are used independently. However, silks and venoms are tightly linked. For example, webs and venom can be used simultaneously to allow spiders to increase the efficiency of prey capture. These physiological adaptations used in conjunction allow spiders to catch prey that can be larger than seven times their own body size (Sanggaard et al., 2014). Similarly, neurotoxins and proteolytic enzymes (similar to those found in the venom from scorpions, wasps and wandering spiders) present in the web silk of golden silk orb‐weaver spiders, *Trichonephila clavipes*, likely function to initially induce paralysis of prey, allowing the spider to reduce venom use for prey capture and manipulation (Esteves et al., 2020). Furthermore, both *Nephila antipodiana* (Zhang et al., 2012) and *Trichonephila clavipes* (Knowlton & Kamath, 2018) use chemical weaponry on their webs to deter predators (myrmicine ants). Another unusual strategy is the ability of spitting spiders (family Scytodidae) to eject fibrous venom on prey (Suter & Stratton, 2009).

However, the production and use of silks and venoms involves a high metabolic cost, and the ways in which silk and venom are used have different outcomes for a spider's fitness (Cooper et al., 2015; Craig et al., 2000). These energetic costs must be balanced according to the prey that is targeted or the risk of predation (Evans et al., 2019). For example, silk production involves protein synthesis, energy consumption, and behavioral costs of web construction (Blackledge et al., 2011). For venom, depletion of venom and changes in venom composition during regeneration could expose spiders to increased risk of predation in a manner similar to that suggested for thick‐tailed scorpions *Parabuthus tranvaalicus* (Nisani et al., 2007). Similarly, newly regenerated venom in the wandering spider, *C. salei,* is characterized by a lower quantity of proteins, and a higher quantity of amino acids, which results in a less acute response in their prey, and could be problematic if the prey is large and difficult to handle (Boevé et al., 1995). These costs can be modulated by adjusting behaviors according to prey availability and type.

The interplay between silk, venom, and individual behaviors related with prey capture, predator deterrence, and ecological factors should be considered to understand the evolution and adaptation of spider weapons, the compensation of the costs associated with these traits, and the optimization of these mechanisms that have allowed spiders to colonize new habitats and adapt to changing conditions. Currently, these relationships are unstudied.

Individual‐level behavioral plasticity and the persistence of behavioral types depends on how adaptive or plastic traits are in response to specific conditions (Bengston & Jandt, 2014). However, behavioral plasticity at the population level may be limited when considering what behavioral types are present in that population (Briffa & Sneddon, 2016; Sih Bell, & Johnson, 2004; Sih et al., 2004). Studying both personality and physiological variables in different species and at different life stages will provide greater insights into how the physiological costs associated with silk production (Craig et al., 1999; Sensenig et al., 2011) and venom production (Evans et al., 2019) might be mitigated through behavioral adjustment (behavioral plasticity).

## 4 CONCLUSIONS

Individual behavioral types are seen in spiders and affect how individuals interact with their environment and ultimately shape behavioral variation at the population level. While it is broadly understood that both extrinsic and intrinsic factors influence the expression of personalities and the levels of plasticity of behaviors, these factors do not act in isolation, and a broader understanding of the interaction between these factors is currently lacking. In spiders, the physiological factors of silk and venom production, both being unique to this group, could offer unique insights into the evolution and ecology of spider personalities because both venom and silk are quantifiable in terms of metabolic costs, can be managed and manipulated by the individual (i.e., a spider can use different types of silks and alter the volume and composition of venom deployed in different situations), affect growth, fecundity and survival of the individual, and may be impacted by hormone expression. To develop a comprehensive understanding of the flexibility of behaviors, and the persistence or absence of behavioral types in spiders, we argue that it is necessary to incorporate these underlying mechanisms into a synthesized whole alongside other extrinsic and intrinsic factors.

## CONFLICT OF INTEREST

The authors declare no conflict of interest.

## AUTHOR CONTRIBUTIONS

**Linda Hernández Duran:** Conceptualization (lead); investigation (lead); writing‐original draft (lead); writing‐review & editing (lead). **David Wilson:** Supervision (lead); writing‐review & editing (supporting). **Mark Briffa:** Writing‐review & editing (supporting). **Tasmin Lee Rymer:** Supervision (lead); writing‐original draft (supporting); writing‐review & editing (equal).

## Data Availability

The paper is a review with no additional data.
